# What Is the Zoo Experience? How Zoos Impact a Visitor’s Behaviors, Perceptions, and Conservation Efforts

**DOI:** 10.3389/fpsyg.2019.01746

**Published:** 2019-07-30

**Authors:** Andrea M. Godinez, Eduardo J. Fernandez

**Affiliations:** ^1^Museology Graduate Program, University of Washington, Seattle, WA, United States; ^2^School of Behavior Analysis, Florida Institute of Technology, Melbourne, FL, United States

**Keywords:** human-animal interactions, zoo visitors, zoo research, visitor perceptions, visitor behaviors, visitor education, conservation

## Abstract

Modern zoos strive to educate visitors about zoo animals and their wild counterparts’ conservation needs while fostering appreciation for wildlife in general. This research review examines how zoos influence those who visit them. Much of the research to-date examines zoo visitors’ behaviors and perceptions in relation to specific exhibits, animals, and/or programs. In general, visitors have more positive perceptions and behaviors about zoos, their animals, and conservation initiatives the more they interact with animals, naturalistic exhibits, and zoo programming/staff. Furthermore, zoo visitors are receptive to conservation messaging and initiatives at zoos and are more likely to participate in on-site conservation opportunities as opposed to after their visits. The research also suggests that repeat visitors are even more inclined to seek out conservation efforts compared to those visiting zoos for the first time. While current research suggests that repeat visitors are more likely to engage in conservation efforts, little is known about causal factors related to such findings, and almost no research exists to-date comparing the conservation efforts of visitors vs. non-visitors. This latter comparison will likely play a greater role in future zoo visitor research, since it poses one of the most important metrics for evaluating the specific effects visiting a zoo can have on people engaging in conservation efforts in general.

## Introduction

Modern zoos have a variety of functions both relative to the species exhibited and the conservation of wildlife in general. According to the Association of Zoos and Aquariums (AZA), some of these goals are: (1) the care and welfare of the animals they exhibit; (2) educating and engaging public, professional, and government audiences; (3) species/habitat conservation; and (4) internal and academic research that increases our knowledge of animals and promotes AZA’s other goals (Reade and Waran, 1996; Fernandez et al., 2009; Association of Zoos and Aquariums, 2013). In addition, zoos have a legacy of being a form of entertainment and are primarily a destination for visitors to attend in their leisure time (Carr and Cohen, 2011). Approximately 700 million people visit zoos and aquariums worldwide annually (Moss et al., 2014), with a 2011 survey indicating that participating zoos and aquariums spent at least $350 million on wildlife conservation internationally (Gusset and Dick, 2011). In a 2012 report by the AZA, 2,700 conservation programs spent approximately $160 million on field conservation for 650 individual species, in addition to ecosystems (Association of Zoos and Aquariums, 2012). It is these high attendance levels and their associated income that gives accredited zoos the ability to fulfill their mission statements.

While zoos are expanding their missions and welcome a large number of visitors, these institutions also have their critics. Animal rights activists and others argue that many zoos contribute little to conservation efforts and also impair zoo animals’ welfare by placing them in captive environments (Hancocks, 2001; Rose et al., 2009; O’Connor, 2010). It is crucial to measure the impact of zoos’ education and conservation initiatives to both indicate the extent of how these organizations are fulfilling their missions and continue to demonstrate the importance of the role of zoos in society despite their critics.

Ultimately, whether an opponent or a supporter of zoological institutions, it is critical to ask: How effective are zoological environments for meeting the welfare, conservation, education, and research goals of accredited zoos? More specifically, what can we learn about how particular captive environments help or hinder these goals? And what can visitors tell us about our ability to successfully meet these goals?

The following paper is a literature review of many peer-reviewed studies that examine how the zoo environment impacts visitors, as well as how these visits impact conservation efforts, both within and outside the zoo. We accomplish this by looking across a variety of disciplines and bodies of work that examine zoological institutions and visitor studies including psychology, museology, animal welfare, and environmental education. Keyword searches of “zoo visitor behaviors,” “zoo visitor perceptions,” “zoo visitor conservation,” “zoo visitor learning,” “animal-visitor interactions,” and other terms occurred in the University of Washington Library’s search engine, in Google Scholar, and in search engines of major publications across these fields. We specifically looked for articles where different factors of the zoo environment (the animals themselves exhibit design, programming/interacting with staff) affected visitor behaviors and perceptions. Articles that examined conservation awareness, attitudes, and behaviors with zoo visitors were also prioritized. In addition, reviewing references cited in relevant articles aided in compiling the studies cited in this literature review. Articles that did not look at visitor learning, post-visit outcomes, or observable zoo visitor behaviors were deemed irrelevant. Specifically, we examine (1) what visitors learn from their zoo experience, with an emphasis on how their behaviors and perceptions are changed and (2) how such visits change those visitors, specifically their conservation efforts. Specifically, we examine how visit frequency affects conservation actions and the need for more research on comparisons between visitors and non-visitors in terms of overall conservation support.

## Discussion

### What Do Visitors Learn at the Zoo?

Zoos are by design an informal learning environment; unless visiting as part of a formal programmatic experience like a school tour, visitors are coming to zoos during their free time and choose which aspects of the zoo they engage with. Visitors to zoos come in with particular motivations like entertainment, bonding time with their families and friends, and also educational experiences (Falk, 2005; Roe and McConney, 2015). For learning to occur, attention is an important pre-cursor for learning (Altman, 1998), as well as connecting with visitors based on their prior knowledge (Dove and Byrne, 2014) and providing entertaining or enjoyable experiences (Spooner et al., 2019).

In order to establish the effectiveness of zoos as a learning environment, it is important to look at a variety of factors that influence visitor learning. Several studies have examined observable behaviors, as well as verbal responses from zoo visitors. These studies have looked at a variety of factors, including the social makeup of visitor groups, educational programming, and the animals in exhibits.

It is also important to understand how visitors cultivate perceptions and attitudes, in addition to studying their behavior, in order to evaluate the effectiveness of a zoo’s education, conservation, and recreation goals (Anderson et al., 2003). Clayton et al. (2009) support the point that educational goals can be improved *via* perceptions. Specifically, positive perceptions can lead to a visitor who is interested in learning more about animals.

#### Effects of the Zoo Environment on Visitor Behaviors

One way to examine a visitor’s response to a zoo exhibit is by measuring observable behaviors displayed by visitors. Specifically, (1) time spent in front of or near an exhibit; (2) attention toward an exhibit (e.g., facing and/or talking about an exhibit); and (3) overall crowd size has been used as measures of interest and satisfaction (Anderson et al., 2003; Margulis et al., 2003; Fernandez et al., 2009; Godinez et al., 2013). Attention is an important measure for visitor studies for which attention can suggest what information visitors are potentially processing and is a precursor to learning (Altman, 1998).

Previous studies suggest that visitor behaviors are influenced by both the presence of a zoo animal and the behaviors it displays. These studies have analyzed and tested the “visitor attraction model”; the theory that active animals attract visitors and have used observable measures such as pointing, stopping, and length of time is facing the exhibit. Results suggest visitors attend more to animal behaviors the more visible and active the animal is and also tend to spend more time in exhibits when an animal is visible and active (Bitgood et al., 1988; Altman, 1998; Anderson et al., 2003; Sellinger and Ha, 2005; Davey, 2006a; Godinez et al., 2013).

Debate over visibility of an animal and its influence on visitor behavior has risen from previous research. Bitgood et al. (1988) found that zoo visitors stopped more often and spent more time at exhibits where the animal was more visible. Whereas Philpot’s (1996) study (as cited in Davey, 2006a, pp. 94–95) found that visitors spent more time searching for animals in naturalistic enclosures, which turned the exhibit and observing animal behaviors into an interactive experience.

In addition to the debate, over animal visibility is the size of the animal. Some studies suggest that visitors prefer larger-bodied animals (Bitgood et al., 1988; Ward et al., 1998). These findings have the potential to influence zoo decisions on the types of animals they display, even considering larger species typically cost more to care for and exhibit. However, Balmford (2000) re-analyzed the results of the Ward et al.’s (1998) study at the Zurich Zoo, which suggested that zoo visitors preferred viewing larger-sized animals. After re-analyzing the data along with additional data collected from the London Zoo, Balmford argued that in terms of visitor length of time at exhibits, there was no discernible difference between time spent at large-bodied animal exhibits and small-bodied animals. Balmford cautions that measures of visitor attention such as time spent attending to an exhibit and crowd size are not necessarily indicators of popularity or preference; smaller animals are typically housed in smaller exhibits, which may make the exhibit itself less appealing, as well as making it difficult for larger visitor groups to form.

Visitor conversations have also been studied in order to examine the influence of animal presence on visitor attention. Altman (1998) analyzed zoo visitor conversations at three bear exhibits as an indirect measure of attention. Conversations were recorded and later categorized as one of four types: (1) animal-directed; (2) human-focused; (3) animal behavior (directed); and (4) other. The study found that animal activity levels appeared to influence visitor conversations, particularly highly animated behaviors. Animal behavior conversation increased and human-related conversation decreased when animals were “highly animated” and the opposite occurred when the animals were pacing or not visible.

Studies examining the impacts of exhibit designs suggest that the transition to naturalistic exhibits in recent decades improves the animal’s well-being as well as visitor behaviors (Nakamichi, 2007; Fernandez et al., 2009). Although the majority of zoo visitors do not interact with signage (Clayton et al., 2009), the context in which an animal is displayed can convey a wealth of information, increase visitor interest, and potentially create a more enjoyable experience. Research also suggests that naturalistic exhibits can increase visitor length of time at an exhibit (Shettel-Neuber, 1988; Davey, 2006a,b). These stay times are constant, even without the presence of an animal (Davey, 2006a; Nakamichi, 2007).

#### Effects of the Zoo Environment on Visitor Perceptions

While interacting with the zoo environment, visitors form perceptions of their surroundings. Previous research argues that zoos can encourage empathy in visitors for the care of zoo animals and, in turn, their wild counterparts and the ecosystems where these animals live. The catalyst for this empathy is positive experiences with animals in zoo environments (Clayton et al., 2009; Kutska, 2009).

Previous studies examining visitor perceptions suggest that perceptions can be influenced and changed by their experiences at zoos. Factors that influence visitor perception can include exposure to and interactions with zoo animals, the exhibit’s design, and elements found within the exhibit space (e.g., signage, enrichment items, and feeding stations), public programming around the exhibit, the ability of visitors to interact with volunteers and staff, and preconceived notions of what certain behaviors (e.g., pacing and other potential stereotypic activity) suggest about the overall welfare of that animal. These aspects have the potential to equally foster or hinder respect and appreciation for zoo animals and the institutions that care for them.

Reade and Waran (1996) conducted a study of how zoo visitors and non-zoo visitors perceived zoo animals in general. The results of this study provided baseline data when examining visitor perceptions across many aspects of zoo operations. The study found that there were significant differences between non-visitors and zoo visitors’ perceptions of animals in zoos. Zoo visitors viewed zoo animals more positively in all questions in the study and thought of them as more attractive, happy, and well-kept. Non-visitors tended to have more negative views of zoo animals across all questions and were significantly more likely to perceive zoo animals as “bored.” In addition, non-visitors also viewed enrichment as less important than zoo visitors. The authors therefore concluded that this difference in perception suggests that the general public is not fully aware of the physical and psychological benefits enrichment has for zoo animals.

Exhibit design also appears to influence visitor perceptions. Zoos have undergone a substantial transformation over the past few decades in exhibit design, with a greater emphasis on naturalistic exhibits, both in terms of their appearance and functionality for the exhibited animals (e.g., ability to hunt and forage). Much of the support for displaying zoo animals in natural contexts is based on behavioral science and theory. In an article about achieving optimal visitor experiences in zoos, Coe (1985) argued that designs, or contexts, of zoo exhibits can reach visitors on both conscious and unconscious levels. These carefully planned contexts can grab the visitor’s attention, and strong multi-sensory exhibit environments have the potential to create strong behavioral responses, such as greater empathy and desire to conserve the exhibited species. This transition to naturalistic exhibits improves visitor perceptions and encourages appreciation and respect for zoo animals (Maple, 1983; Finlay et al., 1988; Reade and Waran, 1996; Nakamichi, 2007).

Visitor perceptions can also be influenced by animal, keeper, and overall exhibit interactions they have while visiting a zoo. When analyzing how visitor perceptions were influenced by small-clawed otter activities, Anderson et al. (2003) found that public animal training and public animal training with interpretation produced more positive zoo experiences and perceptions of exhibit size than passive exhibit viewing or interpretation-only sessions. The educational approach to animal training programming has also been found to be an important factor in influencing visitor learning. A study by Visscher et al. (2009) found that after being told the same facts about Black Rhinoceros during two different types of animal training programs, the school group who received the interpretive presentation (i.e., audience encouraged to ask questions and could touch training tools) answered more post-program questions correctly than the students who attended a less interactive, fact-based presentation. In addition, a study by Lindemann-Matthies and Kamer (2005) found that visitors who attended a staffed “touch table” at a Bearded Vulture exhibit at the Goldau Nature Park and Zoo were more likely to know more about the biology, ecology, and conservation of vultures both immediately after their visit and 2 months post-visit than those who visited the exhibit but only had access to exhibit signage. In addition, educational zoo theater programming performed by staff with no animals present resulted in both children and adult visitors answering more survey questions correctly after attending the performance than answering the same questions before the theater program began (Spooner et al., 2019).

How visitors perceive their experience, as well as the overall welfare of exhibited animals, can be greatly influenced by what behaviors they see the animals engaged in. Captive animal behavior is often broadly defined as positive, healthy behaviors (e.g., searching, foraging, and non-repetitive activity), and negative, “abnormal” behaviors (e.g., hiding, inactivity, and repetitive behaviors, such as pacing). While an operational classification and functional understanding of these behaviors goes beyond the scope of this paper, how such behaviors affect the visitor experience is critical to an overall understanding of what visitors learn at the zoo.

Bexell et al. (2007) examined visitor perceptions of Giant Pandas while playing or not playing. Those who witnessed Giant Panda play were significantly more likely to rate their experience more positively and have a more satisfying experience than those who did not observe playing. As noted previously, Altman (1998) found visitor conversations changed based on bear behaviors, with animal behavior conversations occurring the most when the bears were active compared to pacing and out of sight.

Another factor that influences visitor perceptions of animal behavior is stereotypic activity, broadly defined as repetitive, invariant behavior patterns with no obvious goal or function (Ödberg, 1978; Mason, 1991). In a study by Godinez et al. (2013), the researchers examined how different jaguar behavioral categories correlated with visitor activity and their ratings of the animals’ predominant behavior displayed, well-being, exhibit quality, and the visitor’s enjoyment. Overall, visitors were able to accurately describe a jaguar’s behavior as inactive, active, or out of sight. However, approximately half of all visitors questioned (~47%) defined pacing and other repetitive behaviors as stereotypic, while the other visitors questioned simply described those behaviors as active and non-repetitive. For visitors who described a pacing pattern or other repetitive behaviors as stereotypic, they were also significantly more likely to rate the jaguar’s well-being, exhibit quality, and visitor enjoyment lower than those who described the behavior as non-repetitive, active behavior. Therefore, it appears that acknowledgement of a behavior as a stereotypy can negatively impact multiple perceptions of a zoo visitor’s visit. Similarly, Miller (2013) found that participants rated the overall care of a tiger as lower when the animal engaged in pacing than inactivity. In addition, the participants who observed a tiger pacing were significantly less likely to support zoos after witnessing this behavior when compared to those who observed an inactive tiger. Furthermore, visitors reported have the most positive emotions regarding zoo animals they observed after experiencing up-close animal encounters with animals displaying active behaviors compared to when the animals were out of sight or engaged in other behaviors (Luebke et al., 2016).

While zoos have made significant strides in reducing stereotypic activity displayed by their animals, these studies suggest that public education about such efforts is also necessary. It may be that part of the bias against such stereotypic activity on the part of the observing visitor is due to a lack of knowing what zoos and similar facilities do to deter such activity. Future studies could examine how educating visitors about behavioral enrichment and other welfare-oriented procedures affects their views of exhibited animals, in terms of both how they view the displays of potentially adverse behaviors and how they view the ability of zoos to care for animals.

#### Zoo Visitors Conservation Behaviors

Recent studies have focused on quantifying the effect of zoo visitation on the conservation efforts of those visitors. Most studies to-date have examined a visitor’s conservation knowledge related to a specific exhibit or program before and after interacting with those programs (Hayward and Rothenberg, 2004; Lindemann-Matthies and Kamer, 2005; Lukas and Ross, 2005; Bexell et al., 2007; Chalmin-Pui and Perkins, 2017), as opposed to greater conservation awareness or analyzing a variety of exhibits and programs (Reade and Waran, 1996; Yalowitz, 2004; Falk et al., 2007; Adelman et al., 2010; Moss et al., 2017a,b). Research is emerging to suggest that visitors can have a relatively extensive awareness of human impacts on biodiversity conservation, even when they hold misconceptions regarding concepts about biodiversity and ecosystems (Dove and Byrne, 2014).

When analyzing how zoo visitors respond to conservation efforts within zoos, several studies suggest that one of the most significant factors influencing zoo visitors’ conservation knowledge, attitude, and behaviors is repeat visitation. Repeat visitors retain significantly more conservation information, have more positive attitudes about conservation, and conduct more conservation-related behaviors than visitors who are attending the same zoo for the first time (Yalowitz, 2004; Lukas and Ross, 2005; Miller et al., 2013; Clayton et al., 2017; Moss et al., 2017a). Thus, while we have some knowledge about how repeat visitors differ from first-time visitors, the extent to which this occurs is not known.

In order to evaluate the overall impact zoos may have on increasing visitor interest and activity in conservation efforts, we examine (1) the conservation perceptions, behaviors, and actions taken by the visitor during a given visit; (2) what type of conservation behaviors and perceptions visitors have after their visit; and (3) how do all of these conservation-related efforts differ in zoo visitors compared to those who do not attend zoos.

#### Visitor Conservation Opportunities at the Zoo

*In situ* opportunities for conservation activities provide visitors with a tangible way to contribute to conservation efforts, especially since previous work suggests that visitors are uncertain how to become involved beyond donating money (Ojalammi and Nygren, 2018). On-site conservation activities may also reaffirm conservation behaviors and encourage long-term changes in zoo visitors. When comparing visitors’ conservation actions on-site versus off-site, Stoinski et al. (2002) found that visitors were 20 times more likely to do on-site conservation activities than after their visit to the zoo. Furthermore, facilitating conservation actions *via* staff and programs as opposed to passive visits may increase the potential for visitors to participate in conservation efforts during a visit. In a study conducted during an elephant program at Zoo Atlanta, 350 of 471 visitors studied signed petitions and took solicitation cards. Those who had the highest levels of interaction with the exhibit and elephant program were significantly more likely to return the solicitation cards than those who had lower interaction (Swanagan, 2000).

Another way to encourage *in situ* conservation behaviors is by offering sustainably made items in zoo gift shops, where proceeds go to support conservation efforts (see Sigsgaard, 2009, for a case study of one such effort, and the sustainability issues to consider when stocking souvenirs and other goods in zoo gift shops). An additional on-site conservation action is at the point of admission through the “Quarters for Conservation” program. In this program, the zoo adds 50 cents onto the price of admission and gives their visitors a chance to choose which conservation project they would like their quarter to support. This simple program can help frame the visitor’s entire zoo experience and has been implemented in over a dozen US zoos since the program was founded in 2007 (Hance, 2015).

If zoos continue to strive to demonstrate their effectiveness as conservation organizations, then it is crucial that zoos provide on-site opportunities for their visitors to participate in conservation. *In situ* conservation actions allow zoos to fulfill their missions and demonstrate their impact now. This can also be of great importance when justifying the role of zoos as conservation contributors when critics and others question the effect of zoos on various conservation efforts.

#### Zoo Visitor Conservation Post-visit

When analyzing conservation knowledge retention, some studies have found that visitors’ conservation knowledge and interest persisted after a zoo visit (Jensen, 2014; Moss et al., 2015), but this new understanding rarely results in new conservation actions (Adelman et al., 2010; Miller et al., 2013). However, other studies suggest zoos prompt visitors to rethink their roles in conservation issues after their visit (Falk et al., 2007; Clayton et al., 2017; Jensen et al., 2017). While this is an emerging area of research interest, several studies support that the level and type of engagement with conservation and animals during the zoo experience affect not only visitors’ knowledge retention but also post-visit behavior. Visitors who engaged with films and signage about biodiversity and conservation scored higher on biodiversity knowledge and intent to take part in post-visit conservation actions than those who did not interact with these elements (Moss et al., 2017b). Similarly, a study by Hacker and Miller (2016) indicated up-close encounters with elephants and witnessing active behaviors from the animals had positive effects on visitors’ intent to engage with conservation actions post-visit. In a multi-institutional study of dolphin programs in zoos and aquariums by Miller et al. (2013), participants who witnessed dolphin programs retained much of their conservation knowledge learned from the shows and reported doing more conservation-related behaviors 3 months after witnessing the show than 3 months prior to their visit. Another study examining the effectiveness of touch tables on visitor’s knowledge of bearded vulture biology, ecology, and conservation issues found that visitors who used the touch tables knew more about these items both immediately after their visit and 2-month post-visit than visitors who had not attended the table (Lindemann-Matthies and Kamer, 2005).

In a 2014 study by Jensen analyzing the conservation concerns and conservation self-efficacy of school children both pre- and post-visit, Jensen found an increase in students’ personal concerns about the extinction of species, but marginal differences in if the students felt they could do something about it. Furthermore, a study by Skibins and Powell (2013) suggests that visitors are more inclined to take conservation action for an individual species they connect with, as opposed to conservation of biodiversity on a larger scale. To combat this issue of awareness but lack of action (or widening the impact of said action), others who recommend zoos can take on stronger approaches to motivating visitors to do pro-conservation behaviors that are relevant and easy to implement for a diverse range of zoo visitors (Smith et al., 2012; Grajal et al., 2018). However, providing materials for visitors to participate in post-visit conservation actions has occurred in only a few studies. Analysis that has been conducted to-date suggests that materials that coincide with visitors’ daily lives tend to be more effective in encouraging conservation-related behaviors than those that are less frequent and more in-depth actions. For example, at the Monterey Bay Aquarium, 51% of visitors who picked up a Seafood Watch Pocket Guide tried to use the guide when purchasing seafood after their visit to the aquarium. On the other hand, only 10% of visitors tried to use an “Ocean Allies Card” (a list of conservation organizations to join) after their visit, and no participants joined an organization (Yalowitz, 2004).

#### Zoo Visitors Versus Non-visitor Conservation Actions

To understand fully the degree of impact zoos has on visitors’ conservation efforts, comparisons between zoo visitors and non-zoo visitors are necessary. However, most studies look at zoo visitors alone (Swanagan, 2000; Yalowitz, 2004; Falk et al., 2007; Miller et al., 2013). At least one study to-date indicated that non-zoo visitors viewed zoos as playing an important role in conservation, although non-zoo visitors’ conservation knowledge and attitude were not measured (Reade and Waran, 1996). Because of the importance of comparing differences between zoo visitors and non-zoo visitors to determine the impact zoos have on increasing conservation efforts in general, our final section draws on directions zoos could go in to make such assessments.

## Future Research

Much of the studies done to-date examine changes in visitor behaviors and their perceptions in regard to exhibit design, the presence of animals and their displayed behaviors, and how visitors engage with singular exhibits and/or programs in individual zoos (see “Zoos and Visitors” section of this paper for examples of these studies). This work has laid the foundation for a variety of in-depth questions to be examined moving forward. Specifically, the nuances of how the zoo environment may influence zoo visitors’ appreciation for the animals exhibited, their species’ associated conservation needs, and how the zoo visitors themselves can take conservation actions to support conservation initiatives for the animals’ wild counterparts and their habitats.

As studies continue to examine the conservation impacts zoos have on their visitors, much of the research done to-date can be summarized in an assumed paradigm that zoo visitors go through that are a series of sequential steps with the intended outcome to be conservation-related actions.

**Visit → Knowledge → Concern → Intent → Post-visit action**

However, this paradigm assumes that knowledge is strongly linked to conservation actions. Recent research indicates that other factors like where you live and demographically related factors are more strongly correlated with conservation behaviors than knowledge (Moss et al., 2017a). Based on what studies cited in this literature review indicate, the paradigm could be reframed as follows:

**Visit with *in situ* action → Knowledge → Concern → Intent → Post-visit action**

Given the variety of factors influencing visitors in the free-choice learning environment of zoos and the variety of methodologies used to examine the impact zoos have on their visitors, there is a question beckoned to be asked: *Is it possible to empirically measure the impact zoos have on their visitors?* Many studies mentioned in this review have taken great strides in answering this question—especially when examining how the environment of the zoo (e.g. exhibits and programs) affects behavioral learning and general knowledge of both animal species and the individual animals housed.

Our recommendations are to continue measuring the impact—or to begin measuring the impact—of the following:

1. having a true control group (non-visitors) to understand the full impact zoos may or may not have on zoo visitor knowledge, perceptions, and behaviors;
2. increasing opportunities for on-site conservation activities that visitors can do during their visit; this could potentially improve their conservation knowledge and future conservation actions, as well as be a measurable impact of how zoos are contributing to conservation efforts;
3. providing more opportunities for tangible takeaways for visitors that directly contribute to conservation initiatives post-visit (i.e., Seafood Watch cards, pre-drafted letters to send to legislators, take-home electronic recycling kits) – and then measure the effectiveness of these tools; and
4. studying the phenomena of repeat visitors being more conservation-oriented than one-time visitors. Also begin to study how repeat zoo visitors compare to those who do not visit zoos at all.

On this last point, knowing that research to-date suggests that repeat visitation is a significant factor in conservation knowledge and appreciation for wildlife, we wonder: are repeat visitors continuing to visit zoos because they are already conservation-oriented and see zoological institutions as places to fulfill this area of interest? Or do they become more concerned with conservation issues over time as a result of the information and experiences they have in zoos? Additional studies that delve deeper into motivations of repeat visitors, and how these attitudes and behaviors develop, could shed light on these questions. Regardless of their motivation, these studies suggest that zoos are fostering conservation with this key group of visitors and that those who come to zoos appear to be receptive to and interested in conservation in the first place (Falk et al., 2007).

Zoo membership is a key tool that is readily available to all zoological institutions to help foster the transition from infrequent to frequent visitors. Looking at the motivation, visitors have when signing up for zoo memberships (cost saving, entertainment, interest in animals, interest in conservation, etc.,), and comparing these motivations to conservation-related knowledge, attitudes, and behaviors of members could provide a critical insight into the field.

Although we have described an array of studies for this review, most of them do not address an important aspect to the effectiveness of zoos—how visitors compare to those who do not attend these types of institutions. With the exception of the few studies mentioned earlier in this paper, we have not been able to find peer-reviewed, published research that compares zoo visitors to non-visitors. A plethora of topics, including conservation attitudes, knowledge of wildlife, and other environmental resources, or how these two groups perceive zoos themselves beckons further examination. We suspect that future visitor research will focus more directly on comparisons between zoo visitor and non-visitor conservation efforts, since this is one of the most important metrics for assessing the impact zoos have on increasing the conservations efforts of their visitors, and a necessary measure for evaluating the effect zoos have on the public supporting conservation efforts in general.

## Author Contributions

AG and EF co-wrote and edited the manuscript, as well as researched literature for this review. AG formatted the manuscript in accordance with Frontiers in Psychology guidelines. EF submitted the manuscript for publication.

### Conflict of Interest Statement

The authors declare that the research was conducted in the absence of any commercial or financial relationships that could be construed as a potential conflict of interest.
